# Effects of High Lithium Concentrations on the Growth, Biomass, Mineral Accumulation, Oxidative Stress, Antioxidant and Gene Expression Response, and DNA Methylation in Sunflower Plants

**DOI:** 10.3390/plants15030421

**Published:** 2026-01-30

**Authors:** Francisco Espinosa, Francisco Luis Espinosa-Vellarino, Ilda Casimiro, Carmen Gloria Relinque, Alfonso Ortega, Inmaculada Garrido

**Affiliations:** Research Group of Plant Physiology and Cell and Molecular Biology of Plants, INURA, Faculty of Sciences, University of Extremadura, 06006 Badajoz, Spain; flespinosav@unex.es (F.L.E.-V.); casimiro@unex.es (I.C.); crelrom@unex.es (C.G.R.); aortegagarrido@unex.es (A.O.); igarridoc@unex.es (I.G.)

**Keywords:** antioxidant system, lithium, oxidative stress, reactive oxygen species

## Abstract

This study demonstrates that sunflower plants display integrated, multilevel responses to excessive lithium (Li) exposure. Li concentrations above 5 mM markedly impair germination, growth, and biomass accumulation. Li is preferentially accumulated in the shoots, showing high translocation and bioaccumulation factors, and disrupts mineral nutrient homeostasis, particularly potassium (K) and sodium (Na) uptake, while inducing oxidative stress. Although photosynthetic pigment contents decline, photosynthetic efficiency is largely maintained, except at 10 mM Li. Li treatment enhances superoxide anion (O_2_^.−^) and hydrogen peroxide (H_2_O_2_) production exclusively in leaves. Consequently, activities of superoxide dismutase (SOD), ascorbate peroxidase (APX), dehydroascorbate reductase (DHAR), monodehydroascorbate reductase (MDHAR), and glutathione reductase (GR) increase in leaves, whereas only APX and GR are stimulated in the roots. Nitric oxide (NO) accumulation is detected only in leaves, while hydrogen sulfide (H_2_S) and glutathione (GSH) contents decline. Leaf ascorbate (AsA) levels decrease concomitantly with dehydroascorbate (DHA) accumulation. Expression analyses of catalase, DHAR, DHAR-like, and glutathione S-transferase (GST) genes confirm their involvement in Li stress responses. Moreover, global DNA methylation analyses reveal hypomethylation in leaves and hypermethylation in the roots. Overall, Li exposure induces dose- and organ-specific physiological, molecular, and epigenetic adjustments in sunflower plants under environmentally relevant concentrations and controlled experimental conditions in this study.

## 1. Introduction

Lithium (Li) is a key strategic resource in the global energy transition, primarily due to its use in rechargeable batteries for electric vehicles, electronic devices, and renewable energy storage systems. It is an element with increasing demand due to the rise of these technologies, which has led to intense geopolitical competition for its control. Furthermore, recent reports such as that by the International Energy Agency (2024) [1] warn that current projects will only cover 50% of the global Li demand by 2035, highlighting the urgent need for investment and planning in order to ensure the supply for the future. Also, it is essential to study the consequences that high levels of Li, caused by anthropogenic activities, may have on plants [2]. Li is widely distributed, in small quantities, throughout the Earth’s crust. While geogenic Li is poorly soluble, when added to soil (contamination), it is one of the most mobile cations and can seep into groundwater and reach surface water through runoff, thus potentially affecting crops. Li is easily absorbed by plants and is therefore able to enter the food chain [3].

Although Li is absorbed by plants, it does not appear to be necessary for their growth, and it is not considered to be an essential element since it is not required to complete their life cycle. Plant Li content is a good indicator of soil Li status. However, plants show considerable differences in their ability to absorb and tolerate this element, and the levels at which it exhibits negative effects also vary [4,5]. Some families, such as *Solanaceae*, *Ranunculaceae*, and *Rosaceae*, tend to accumulate high levels of Li in their tissues. *Lycium* (*Solanacea*) is capable of accumulating considerable amounts of Li even when it is not abundant in the soil [4,6]. However, there is considerable evidence that Li at low concentrations stimulates growth in certain plants and may have metabolic functions in halophytes; despite this, at high concentrations it is toxic to all plants [7,8]. The low toxicity of Li has sometimes led to it not being considered as an emerging environmental contaminant. Li may be beneficial in some cases; however, the effect of prolonged and high exposure is unknown [9]. The amount of Li in plants typically ranges from 0.2 to 30 ppm, depending on how species uptake or reject Li [10].

Li accumulation in plants depends on several factors, such as the Li content in the soil, the clay content and pH of the soil, the plant’s ability to accumulate it, and the coexistence of other ions with similar physicochemical properties in the soil [4]. Moreover, Li can also alter the absorption of other mineral elements. Thus, decreases in the absorption of potassium (K), calcium (Ca), and magnesium (Mg) have been noted [4,11,12]. Li seems to have an antagonistic effect with Ca, rubidium (Rb), and probably with zinc (Zn), and synergistic effects with iron (Fe) and manganese (Mn) [4]. Furthermore, Li, in both plants and animals, interacts with sodium (Na), K, as well as with the enzymes that require Mg [10].

The Li transport mechanism appears to be the same as the K transport systems of the plasmalemma, such as low-affinity cation transporters, a non-selective cation channel (NSCC), and high-affinity K transporters [13]. Regarding its distribution in cells, Qiao et al. [14] showed that up to 29% of Li is in the cell wall of the Li accumulator *Apocynum venetum*, although most of it accumulates in the vacuole (up to 72%). Once Li is absorbed, it can be translocated to other areas of the plant and accumulate in the roots [8] or leaves [4,5,7,15].

Li has a detrimental effect on plant growth and biomass production [15,16,17]. It can affect the photosynthetic pigment content and the photosynthesis level, as well as other metabolic paths [6,7,15]. Its interaction with other mineral elements can alter ionic homeostasis [13,18]. These Li toxic effects may be related to the development of oxidative stress, altering the cellular redox homeostasis and potentially inhibiting antioxidant enzymes [19]. This oxidative stress might also affect the transcription of genes responsible for antioxidant production. Different results have been obtained because this response appears to be organ- and species-dependent [5].

The present study examines how a critical mineral such as lithium (Li) affects processes including germination, growth, accumulation, and essential nutrient content, as well as photosynthesis and the production of reactive oxygen species (ROS), reactive nitrogen species (RNS), and reactive sulfur species (RSS). It also analyzes the activity and molecular regulation of enzymatic and non-enzymatic antioxidant systems triggered by the presence of different Li concentrations in an agronomically important crop such as sunflower (*Helianthus annuus* L.). Furthermore, in light of the numerous studies highlighting the role of epigenetic mechanisms, such as DNA methylation, in plant defense against abiotic stresses, including salinity, drought, and exposure to heavy metals or metalloids [20,21,22,23,24,25], the level of cytosine methylation—a reaction catalyzed by DNA methyltransferases—is evaluated [26,27,28,29]. Overall, this work aims to provide a deeper understanding of the effects of lithium on plants, using sunflower as a model crop, by assessing dose-dependent responses. Mainly, it considers evidence showing that low Li concentrations may promote germination and plant development, whereas higher toxic doses induce stress responses and activate specific defense mechanisms of greater relevance.

## 2. Results

### 2.1. Germination, Growth, Development, and Biomass Production

**Figure 1:**
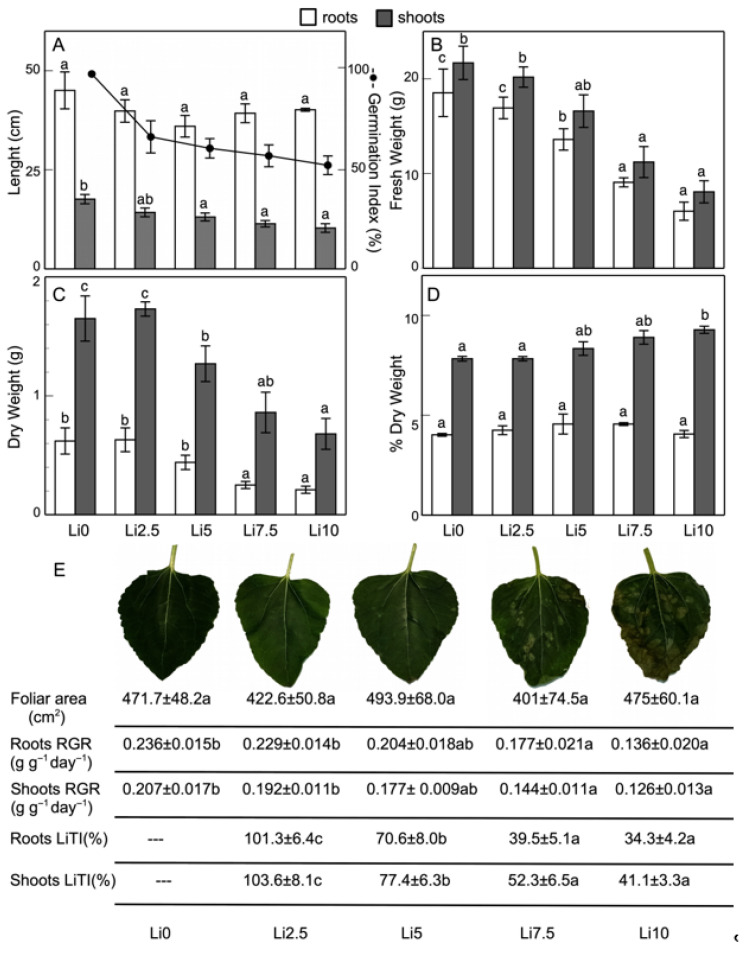
Effect of Li on (**A**) germination index and length (l), (**B**) fresh weight (FW), (**C**) dry weight (DW), (**D**) %DW in roots and shoots (**D**), and (**E**) leaves and foliar area, relative growth rate (RGR), and Li tolerance index (LiTI) in roots and shoots of sunflower plants. Values are means, error bars denote ± standard deviation (n = 15), and different letters indicate a significant difference among treatments of the same plant organ *p* < 0.05.

Figure 1A shows the effects that the different Li concentrations used have on the germination of sunflower seeds, expressed as the germination index (GI%), and the root and shoot lengths of sunflower plants. As can be seen in sunflowers, an increase in the Li concentration in the environment reduces the GI%, from 71.7% (I = 32.1) for Li2.5 to 54.6% (I = 46.63) for Li10. Regarding the root length, no significant growth alterations are observed at the different doses of Li used. As can be seen, very similar values are obtained for all concentrations when compared to Li0, although there is a small but not significant decrease in the root length (approximately 12% for all concentrations, except for Li5, which reaches 20%). The growth of shoots is more sensitive to Li, with decreases in the shoot length ranging from 19% for Li2.5 to 41% for Li10, compared to the value observed for Li0.

At the root and shoot levels, fresh weight (FW) (Figure 1B) is significantly reduced at the highest concentrations (9.08 mg and 6.02 mg in the roots and 16.59 mg and 11.21 mg in the shoots, for Li7.5 and Li10, respectively), but not at the lowest two concentrations, when compared to Li0 (18.52 mg and 21.67 mg, respectively). These decreases are similar in the roots and shoots, ranging from 8% in Li2.5 to 65% in Li10. The same behavior with Li doses is observed in dry weight (DW) (Figure 1C). The decrease in DW is somewhat greater in the roots than in the shoots, with a negative correlation. Thus, in Li10, root DW decreases by 66%, whereas in the shoots the decrease does not reach 60%. Li does not significantly alter the DW/FW ratio (Figure 1D) in the roots. By contrast, this value increases significantly in the shoots, from 7.83 in Li0 to 9.28 in Li10. Regarding the appearance of visual symptoms of leaf alterations (Figure 1E), only Li7.5 and Li10 show chlorotic areas and, at the highest concentrations, even necrosis at the edges. The roots of plants that were grown in Li show slight darkening. However, the total leaf area is not significantly altered with increasing Li concentration in the growing medium (Figure 1E), but it does affect shoots RGR and roots RGR, which decrease at the highest Li treatments (Li7.5 and Li10), while with the lowest used, Li2.5, it is not significantly affected, compared to control values (Figure 1E). Thus, shoots RGR decreases in Li7.5 and Li10, reaching the lowest values in Li10 (61% with respect to Li0). By contrast, roots RGR is not altered in Li2.5 and decreases in the rest of the treatments (27% and 50% less growth for Li7.5 and Li10, respectively). With regard to the LiTI (Figure 1E), it can be seen that an increase in the amount of Li causes a decrease in the LiTI, ranging from 101.3, 70.6, 39.5, and 34.3% for roots LiTI and 103.6, 77.4, 52.3, and 41.1% for shoots LiTI, for Li2.5, Li5, Li7.5, and Li10, respectively. The LiTI is similar in both organs, although it shows greater sensitivity in the roots than in the shoots. This index is not affected by lower Li concentrations (Li2.5), and higher concentrations show a greater decrease with very similar values.

### 2.2. Li Content and Other Mineral Elements

**Figure 2:**
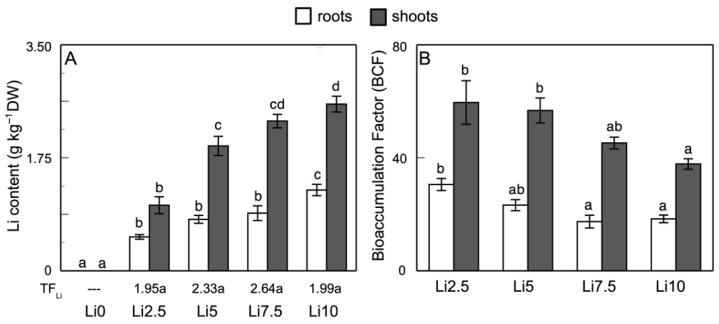
Effect of Li on (**A**) Li content and translocation factor (TF_Li_), and (**B**) bioaccumulation factor (BCF_L_) of Li in roots and shoots of sunflower plants. Values are means, error bars denote ± standard deviation (n = 15), and different letters indicate a significant difference among treatments of the same plant organ *p* < 0.05.

Regarding Li uptake, accumulation, and transport, a dependence is observed both on the amount of Li to which the plants are exposed and the measured organ (roots or shoots). Figure 2A shows that the amount of Li accumulated in the roots increases from 596 mg Li kg^−1^ DW (Li2.5) to 1434.5 mg Li kg^−1^ DW (Li10). In the shoots, the total Li content increases from 1162.7 mg Li kg^−1^ DW in Li2.5 to 2950.9 mg Li kg^−1^ DW in Li10 (2.6 times). The total Li content per plant depends on the external concentration, which reaches a maximum value of 2.56 mg plant^−1^ in Li10.

Li accumulation occurs primarily in the shoots. This high accumulation is reflected in the TF_Li_ values (Figure 2B), with values of 1.57, 2.08, 2.38, and 1.89 for Li2.5, Li5, Li7.5, and Li10, respectively. Regarding the BCF_Li_ values, a significant decrease is observed with increased concentration, from 34.4 to 20.7 in the roots and 67.0 to 42.5 in the shoots, for Li2.5 and Li10, respectively. The total amount of accumulated Li is greater in the shoots than in the roots and increases depending on the external Li concentration.

**Figure 3:**
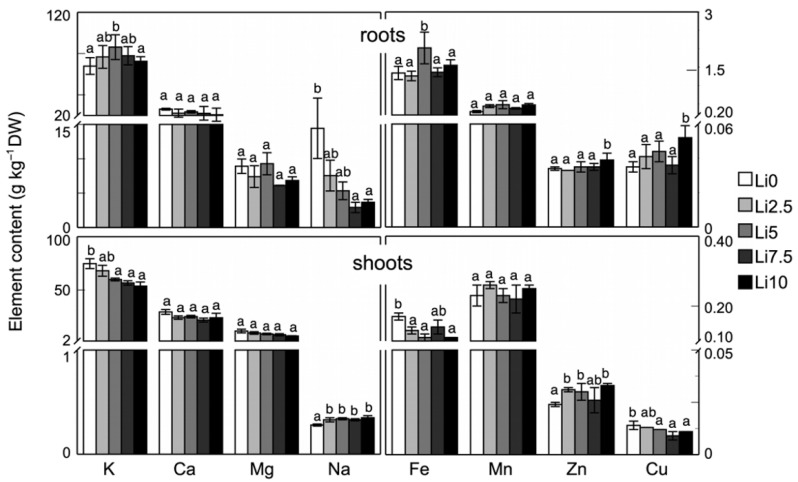
Effect of Li on mineral element contents in roots and shoots of sunflower plants. Values are means, error bars denote ± standard deviation (n = 15), and different letters indicate a significant difference among treatments of the same plant organ *p* < 0.05.

The mineral composition of sunflower plants is influenced by exposure to Li (Figure 3). While elements such as Ca, Mg, and Mn do not show any noticeable changes in their absorption, transport, or accumulation in shoots and roots, other elements—including K, Na, Fe, Zn and Cu—do exhibit alterations. In the case of K, Fe, and Cu, a similar trend is observed: an increase in absorption and accumulation in the roots, accompanied by a decrease in the shoots. However, Na displays an opposite pattern to these previous three elements, as its absorption decreases in the roots while increasing in the shoots. Zn exhibits a distinct behaviour compared with the other, showing an increased presence in both shoots and roots.

### 2.3. Photosynthetic Pigment Content and Photosynthetic Efficiency

Chlorophyll *a* and *b* contents and carotenoid content (Table 1) decrease with increasing external Li concentration. The decrease in Chl *b* content is somewhat greater than that in Chl *a* and carotenoid contents. In Li2.5, the Chl *a* content decreases by 7% (not significant), whereas the Chl *b* content decreases by 13% and the carotenoid content by 6%. However, in Li7.5 and Li10, the decrease in all of the pigment contents is very similar and more significant, ranging between 25% and 33% in all of them. The Chl *a/b* and carotenoids/Chl ratios are not altered in response to Li treatment.

Regarding the photochemical activity of chloroplasts (Table 1), no alterations in the efficiency of the photosystem II (F_V_/F_M_) due to Li are observed, except for Li10. F_0_ values do not undergo significant changes compared to control values. F_V_/F_0_ decreases only in Li10 (3.49 vs. 4.54 in Li0).

### 2.4. Effect on Lipid Peroxidation and Protein Carbonylation

**Figure 4:**
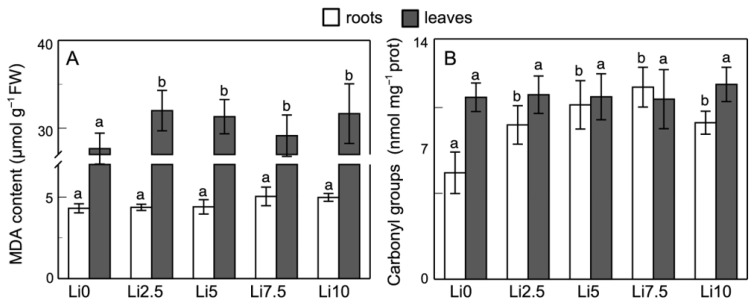
Effect of Li on (**A**) lipid peroxidation (MDA content), and (**B**) carbonyl groups in roots and leaves of sunflower plants. Values are means, error bars denote ± standard deviation (n = 15), and different letters indicate a significant difference among treatments of the same plant organ *p* < 0.05.

Figure 4 shows the results for lipid peroxidation and protein carbonylation, biomarkers of oxidative stress, for the different Li treatments. As can be seen in sunflower roots (Figure 4A), MDA production in Li2.5 and Li5 is similar to control values. In treatments with higher amounts of Li, an increase is observed, although it is not significant. Regarding the leaves, the amount of MDA shows an upward trend, although it is not significant, in all of the treatments (≈15% in response to Li).

Regarding the effect of Li on protein carbonylation (Figure 4B), the results show a significant increase in the roots, with no alteration in leaves. For Li0, a value of 6.2 nmol mg^−1^ protein is observed, which increases by 45%, 64%, 80%, and 47% for Li2.2, Li5, Li7.5, and Li10, respectively. This response shows that protein carbonylation in the roots increases with external Li concentration but decreases at the highest concentration used (Li10).

### 2.5. Effect of Li on the Contents of Reactive Oxygen Species, Reactive Nitrogen Species, and H_2_S

**Figure 5:**
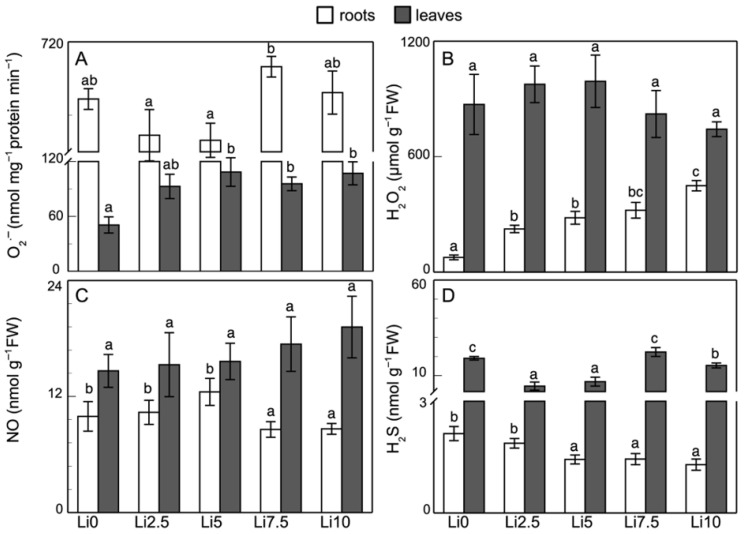
Effect of Li on (**A**) O_2_^.−^ production, (**B**) H_2_O_2_, (**C**) NO, and (**D**) H_2_S contents in roots and leaves of sunflower plants. Values are means, error bars denote ± standard deviation (n = 15), and different letters indicate a significant difference among treatments of the same plant organ *p* < 0.05.

**Figure 6:**
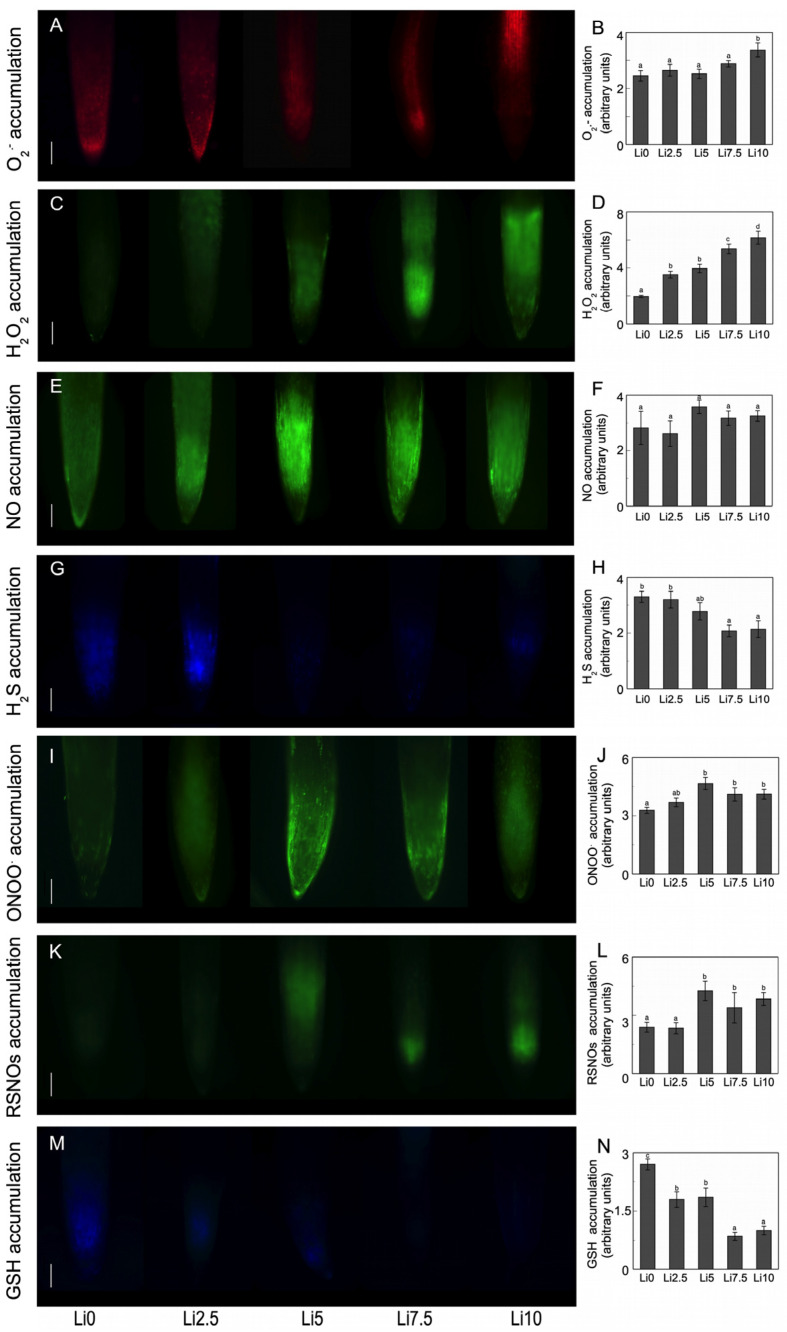
Micrographs of longitudinal sections and the average fluorescence intensity levels quantified in arbitrary units in a longitudinal section of the primary roots for (**A**,**B**) O_2_**^.−^**, (**C**,**D**) H_2_O_2_, (**E**,**F**) NO, (**G**,**H**) H_2_S, (**I**,**J**) ONOO**^−^**, (**K**,**L**) RSNOs, and (**M**,**N**) GSH in sunflower exposed to Li treatments. Scale bar, 200 µm. Values are means ± standard error, and different letters indicate a significant difference among treatments at *p* < 0.05. At least 10 roots were tested for each treatment and 5 independent repetitions were analyzed.

O_2_^.−^ production (Figure 5A) in sunflower roots is not significantly altered. In leaves, there is an increase in O_2_^.−^ production, independent of the Li concentration used. Thus, it increases from 50.6 nmol O_2_^.−^ mg^−1^ protein min^−1^ to 107.0 nmol O_2_^.−^ mg^−1^ protein min^−1^ for Li0 and Li10, respectively. Regarding the H_2_O_2_ content (Figure 5B), it is observed that Li causes a significant increase in the H_2_O_2_ content in the roots. This content is higher when more Li is present in the environment (194% × 2.9, 270% × 3.7, 321% × 4.2, and 488% × 5.9-fold, for Li2.5, Li5, Li7.5, and Li10, respectively). By contrast, Li has no effect on the H_2_O_2_ content in leaves. These effects of Li on ROS were visualized at the level of the roots in vivo using specific fluorescent probes. Thus, it can be observed that there are no significant differences in O_2_^.−^ production (Figure 6A,B) and accumulation due to the effect of the Li treatments, but there is a sharp increase in the H_2_O_2_ content (Figure 6C,D).

Regarding the NO content (Figure 5C), it is observed that in the roots there is only a 26% (1.26-fold) increase in the NO content for Li5, with no significant changes in the remaining treatments. However, in leaves, the NO content depends on the Li concentration, with increases of 20% and 31% for Li7.5 and Li10, respectively, with no change at lower concentrations. Figure 6E,F, Figure 6I,J, and Figure 6K,L show the accumulations of NO, ONOO^−^, and RSNOs, respectively, in response to Li in the roots. Meanwhile the NO content is not altered, and the ONOO^−^ and RSNOs contents increase significantly in response to Li-induced stress.

Li induces a decrease in the H_2_S content (Figure 5D and Figure 6G,H). In the roots, this decrease becomes significant starting at Li5, with decreases in the H_2_S concentration from 32 to 39%. In leaves, the content also decreases, but only in Li2.5 and Li5, with H_2_S contents similar to control values in Li7.5 and Li10.

### 2.6. Antioxidant Enzyme Activities and Components of the AsA-GSH Cycle

**Figure 7:**
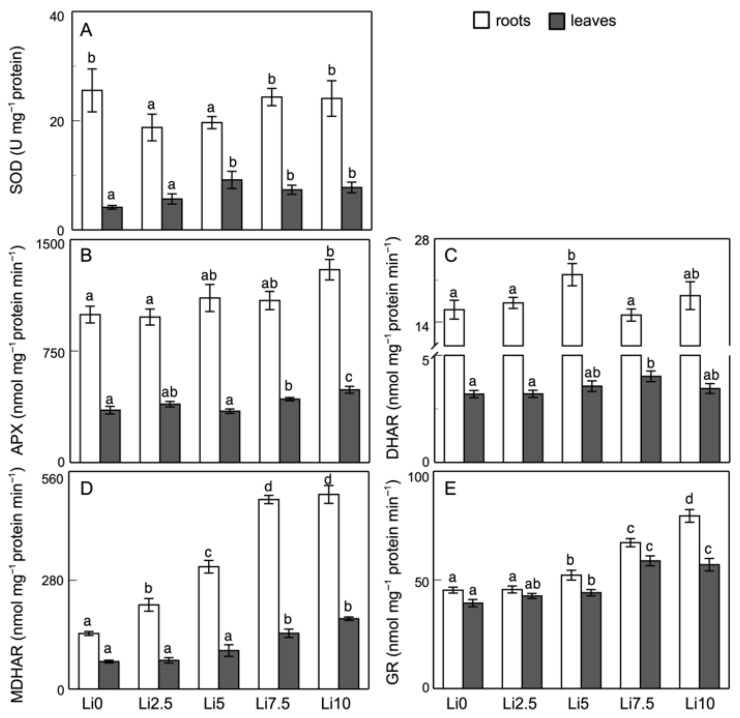
Effect of Li on antioxidant (**A**) SOD, (**B**) APX, (**C**) DHAR, (**D**) MDHAR, and (**E**) GR activities in roots and leaves of sunflower plants. Values are means, error bars denote ± standard deviation (n = 15), and different letters indicate a significant difference among treatments of the same plant organ *p* < 0.05.

Regarding the enzymatic activities of SOD, APX, DHAR, MDHAR, and GR (Figure 7), an alteration in their function is observed in response to Li. The SOD activity (Figure 7A) in the roots increases significantly in Li2.5 (29%) and Li5 (44%), whereas it decreases at the two highest concentrations. In leaves, this activity is not affected by Li.

Regarding APX activity (Figure 7B), an increase can be seen in the roots starting from Li5, although this increase is only significant in Li10 (a 30% increase). In leaves, the increase in this activity begins to be significant starting from Li7.5 (increasing by 21% and 39% at Li7.5 and Li10, respectively). DHAR activity (Figure 7C) is activated by Li in both the roots and leaves, although this activation is only clearly significant at a single Li concentration (37% in the roots in Li5, 22% in leaves in Li7.5). On the other hand, MDHAR activity (Figure 7D) appears to be the most sensitive to Li, with strong increases in activity in both the roots and leaves, and is dependent on the Li concentration, especially in the roots, with increases, all of them significant, of 52%, 120%, 241%, and 251% for increasing Li concentrations. In leaves, activation of this enzyme is significant only at the two highest concentrations, with increases of 103% and 156% for Li7.5 and Li10, respectively, relative to Li0. Finally, GR (Figure 7E) shows a very similar response to MDHAR, with greater increases in its activity in the roots than in leaves, which are significant starting at Li5.

**Figure 8:**
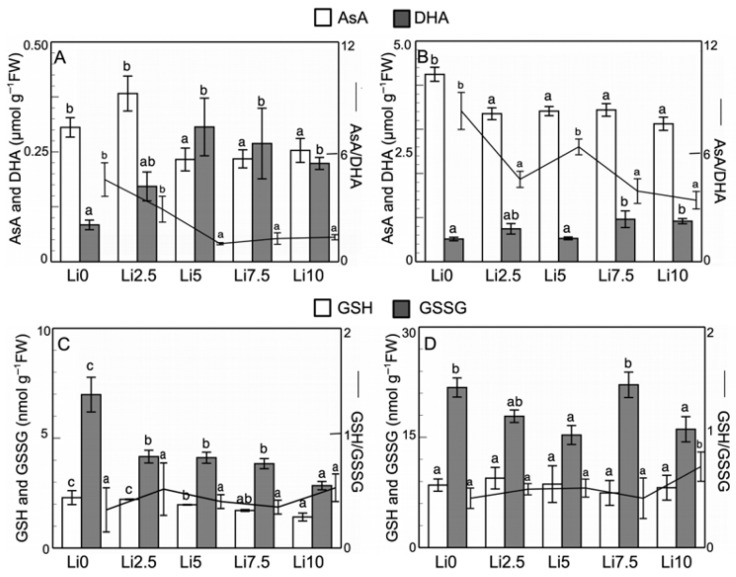
Effect of Li on AsA–GSH cycle components: (**A**,**B**) AsA, DHA, and AsA/DHA ratio, and (**C**,**D**) GSH, GSSG, and GSH/GSSG ratio in roots and leaves of sunflower plants. Values are means, error bars denote ± standard deviation (n = 15), and different letters indicate a significant difference among treatments of the same plant organ *p* < 0.05.

Figure 8A,B shows the AsA and DHA contents for both the roots and leaves, respectively, and the AsA/DHA ratio. Compared with the control values (Li0), Li treatments induce a decrease in the AsA content and increase in the DHA content in both the roots and leaves. In the roots, the greatest effect on the AsA content is observed starting at Li5, with values that decrease by around 20% relative to Li0. The DHA content increases in all of the treatments, with a maximum increase in Li5 (3.7-fold). In leaves, the decrease in AsA content is similar in all of the treatments (≈20%), with increases in the DHA content at all concentrations except Li5. Li2.5 induces a 1.45-fold increase in the DHA content, while Li7.5 and Li10 show ≈1.85-fold increases. The total pool in the roots is not affected, and in leaves it decreases significantly in Li2.5 and Li5 (due to the decrease in AsA content since the DHA content is practically unchanged at these concentrations). In Li7.5 and Li10, both forms of ascorbate compensate for each other, with the total ascorbate content remaining unaffected when compared to Li0. In comparison to the control values, the AsA/DHA ratio decreases in both the roots and leaves. In the first case, the decrease becomes significant starting at Li5, and in the second, a significant decrease is observed in Li2.5, Li7.5, and Li10, but not in Li5. In general, the decrease is greater in the roots than in leaves; thus, in Li10, it is 76% in the roots versus 60% in leaves, compared to Li0.

The GSH and GSSG contents in the roots and leaves are shown in Figure 8C,D, respectively, and the GSH/GSSG ratio. The GSH and GSSG contents decrease in the roots in response to the increase in stress induced by the Li concentrations. The decrease in GSH levels in the roots is also evident with fluorescence microscopy (Figure 6M,N). By contrast, the GSH content is not altered in leaves, although an increase is observed in Li2.5 and slight but not significant decreases are observed in Li7.5 and Li10. The GSSG content decreases in all cases except in Li7.5, where similar values to the control are obtained. As a result of these changes, the total pool in both organs decreases, with the lowest value in Li10 roots (45%) and in Li5 leaves (28%). Li-induced stress causes the GSH/GSSG ratio (Figure 8C,D) to generally increase in both organs, although this increase is not significant in any case.

### 2.7. Altered Expression of CAT, DHAR, DHAR_like, and GST Under Li Stress

**Figure 9:**
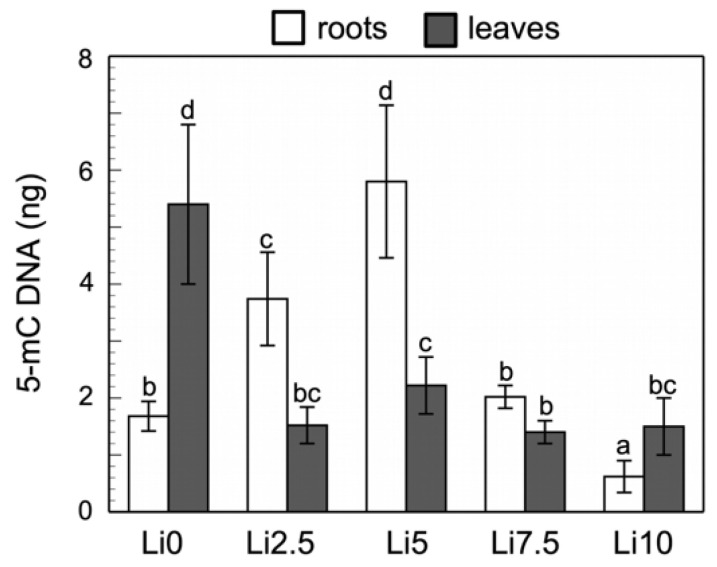
Effect of Li on the levels of CAT, DHAR, DHAR_like, and GST gene expression in roots and leaves of sunflower plants. Values are means, error bars denote ± standard deviation (n = 15), and different letters indicate a significant difference among treatments of the same plant organ *p* < 0.05.

After quantifying the expression of *CAT*, *DHAR*, *DHAR_like*, and *GST* (Figure 9), the results show that *CAT* expression increases as stress (Li dose) increases at the leaf level. However, this trend is not true in the roots in Li10, as the expression level of this enzyme declines, although it remains higher than in the unstressed root. *DHAR* has an expression pattern in leaves that is characterized by a decrease in its expression as the Li content increases, although the opposite occurs in the roots: higher stress indicates a higher expression of this enzyme, except in Li2.5. *DHAR_like* has a different expression pattern at the leaf level because its expression increases up to Li5, and then declines, the same as in the roots, where the highest expression level is reached in Li7.5, and then it decreases to values similar to Li5. The highest expression levels occur in the roots. Regarding *GST*, its expression pattern is similar to that of *DHAR_like*, with higher expression in the roots than in leaves, and a pattern that increases from control plants with increasing Li concentration, up to Li10, where the expression decreases (50% of the Li7.5 value).

### 2.8. Quantification of Global DNAg Methylation

**Figure 10:**
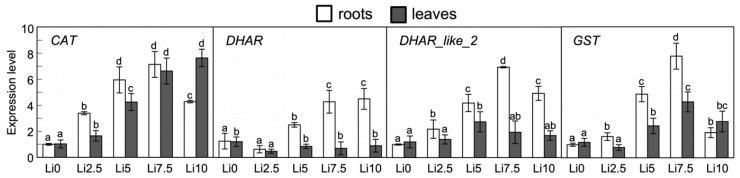
Effect of Li on the methylation level of cytosines in roots and leaves of sunflower plants. Values are means and error bars denote ± standard deviation (n = 15), and different letters indicate a significant difference among treatments of the same plant organ *p* < 0.05.

Figure 10 illustrates that cytosine methylation is higher in the roots than in leaves of sunflowers under Li stress, except in the Li10 samples. In leaves, a significant decrease in methylation is observed in response to Li toxicity. By contrast, in the roots, significant hypermethylation is detected in Li2.5 and Li5. From Li7.5 onward, cytosine methylation declines, reaching hypomethylation in Li10.

## 3. Discussion

The germination-inhibiting effect of Li on *H*. *annuus* seeds was very similar, albeit at lower Li doses (Figure 1A), to that previously described by Kavanagh et al. [17], who tested 34 different species and observed a decrease in germination in response to increased Li concentrations in most of them. However, these authors described that, together with *Brassica napus*, *B*. *oleracea*, and *Solanum lycopersium*, *H*. *annuus* maintained high germination levels (90%). Gayathri et al. [30] and Kuloğlu et al. [31] described the inhibitory effects of Li on the germination of *Amaranthus viridis* and *Allium cepa*. Li et al. [21] for *B*. *carinata*, Jiang et al. [32] for *Apocynum pictum*, and Iannilli et al. [33] for *Lepidium sativum* described increases in germination at low Li concentrations, with a decrease at higher concentrations. Da Silva et al. [34] obtained higher germination percentages in soybean seeds by applying different doses of Li (sulfate and hydroxide).

Regarding the lengths of the root and shoots (Figure 1A), there was no significant change in the root length, although a downward trend can be observed. The shoot length showed a significant decrease. These results were consistent with those obtained by Li et al. [21], who, although they described a reduction in the root length with an increase in the Li concentration (up to 180 mM LiCl) in *B*. *carinata*, this did not occur at concentrations similar to those used in the present study (up to 10 mM LiCl), where no significant changes were observed. Jiang et al. [15] described for *A*. *venetum* that Li negatively affected growth. This negative effect has been described by other authors, such as Sattar et al. [35], who reported that LiCl reduced the growth of potato plants. The FW decreased in both the roots and shoots with an increase in the Li concentration (Figure 1B), and these results coincided with those obtained by other authors [7,8,21,36]. However, Gayathri et al. [30] indicated that exposure to low Li concentrations increased the size while high concentrations increased the biomass of *A*. *viridis* plants. DW decreased in sunflowers with an increase in Li exposure, more markedly in the roots than in the shoots (Figure 1C), and this coincided with the results obtained by Macedo et al. [37] for *Ricinus communis*. While in the roots DW/FW was not altered, in the shoots it increased. Kalinowska et al. [8] observed a higher water content in lettuce after exposure to low Li concentrations, possibly due to an effect of Li that increases the water adsorbed by macromolecules, although at high concentrations it competes with water, acting as a dehydrating agent. This effect could be responsible for the increase in the DW/FW ratio (Figure 1D) observed in sunflower shoots, where a large accumulation of Li occurs. The reduction in water content is a toxic effect of Li that reduces turgor and growth at high concentrations, but it is unclear whether this is due to decreased water uptake or increased transpiration [18,38]. Reductions, similar to those found in the present study, in biomass production, growth, FW and DW, roots RGR, and shoots RGR (Figure 1E), the appearance of necrotic spots on the leaves, and root browning due to different doses of Li have been described for several species [8,18,31,39]. Furthermore, high Li treatments (Li7.5 and Li10) resulted in lower roots LiTI and shoots LiTI (Figure 1E), indicating a toxic effect at these concentrations. Similar results were described in *Amaranthus viridis* [30], where the tolerance index decreased after 21 days of Li treatments, but this index increased over a longer period. Our results were similar to the behavior described for heavy metal toxicity [40].

The absorbed Li accumulated mainly in the shoots (Figure 2A). This higher accumulation in the shoots and its dependence on the Li supply has been previously described [7,15,41,42]. By contrast, in lettuce, Kalinowska et al. [8] observed a preferential accumulation of Li in the roots. The BCF values (Figure 2B) decreased with an increase in the external Li concentration, although in all cases with high values. This seems to indicate a higher capacity for Li bioaccumulation when the plants are subjected to low concentrations of this element. The high BCF values showed the great capacity of sunflowers to absorb and accumulate Li in their tissues, especially in leaves [17], similar to the effect observed and described by Török et al. [43], which was also experienced in a hydroponic environment, as well as by Hawrylak-Nowak et al. [7]. The decrease in BCF values with the increase in external Li concentration was similar to that described for high concentrations in *Apocynum* [32,39], soybean [42], *S. natans* [43], and *Lolium* sp. [44]. TF_Li_ (Figure 2A) increased with an increase in the Li content in the external environment up to Li7.5, decreasing in Li10. TF_Li_ was shown to be dependent on the Li concentration in the environment. This behavior coincided with the results observed by Török et al. [43] for *S. natans*, and partially with those described by Shakoor et al. [42] and Jiang et al. [32], with an increase in TF values in response to increased external Li concentrations and decreasing at high concentrations. Our results indicated that TF_Li_ correlated directly with exogenous Li concentrations in the culture medium, except for Li10, which showed a value similar to Li2.5.

The results obtained regarding K content (Figure 3) coincided with those described for spinach, canola leaves, and *S. natans* [18,41,43]. Hawrylak-Nowak et al. [7] described that, for sunflowers, low Li concentrations did not alter the leaf K content, but it was altered when grown with 50 mg L^−1^ Li, in which case it increased. By contrast, Törok et al. [43] described a decrease in the K content of the aquatic plant *S. natans*, as did Shah et al. [12] for potato calluses. Decreases in Mg content have been described by several authors [12,43]. A similar behavior was observed with the higher concentrations of Li used in our case, although the change was not significant. Regarding Ca, the results were highly variable. Thus, Bakhat et al. [41] did not obtain any alteration in the Ca content in the roots, but in leaves a decrease was observed with an increase in Li content, which coincided with our results in the roots. On the contrary, Törok et al. [43] showed an increase in the Ca content induced by Li, whereas the Na content only decreased at high concentrations but not at lower ones. This behavior was similar to that obtained in the present work in the roots, but not in leaves, where their content increased. Also, Dawood et al. [18] described an increase in the Na content due to the effect of Li. Törok et al. [43] described decreases in the Fe, Mn, Zn, and Cu contents. These results were consistent with ours for the Fe and Cu contents in leaves, while they showed an opposite behavior for Mn and Zn contents. The effect of Li on this process is unclear, but Li competition with these mineral elements for uptake and transport systems could be involved. Li interacts with other elements, such as K, Na, and Mg [10].

According to previous studies, Li enters plants through the roots via passive influx through non-selective cation channels (NSCCs), which also mediate the transport of Na and K [45,46]. Consequently, competition among these elements for cellular entry would be expected to reduce Na and K accumulation in Li-exposed plants. In the case of K, a decrease was observed exclusively at the foliar level, whereas no significant alterations were detected in the roots. By contrast, Na accumulation decreased in the roots in the presence of Li, which is consistent with competition between both ions for shared entry pathways. However, at high Li doses, an increase in foliar Na content has been reported. Although Na is not an essential nutrient, it participates in key physiological processes, including stomatal regulation, osmotic balance, and metabolic functions. Under Li-induced stress, Na may therefore be rapidly translocated to the shoots, preventing its accumulation in the roots. In addition, the increase in foliar Na may reflect its partial functional substitution for K, which is less abundant in leaves under this stress condition, as both ions contribute to the regulation of stomatal movement, plant water, and osmotic status.

Regarding Mg, competition with Li at the level of root entry did not occur, unlike that observed for Na and K. Nevertheless, Li exhibits a high affinity for the Mg binding sites of specific transporters [47,48,49,50] owing to the similarity of their ionic radii [51], despite their different charges. This interaction would theoretically reduce Mg uptake through competitive inhibition; however, no significant differences were observed in Mg uptake or accumulation. Although Li does not appear to impair Mg acquisition, it may inhibit Mg-dependent enzymes such as inositol monophosphatase (IMPase) [48,49], which is essential for growth, signaling, and cellular osmoregulation. As a result, Li exposure may lead to reduced vegetative growth at both the shoot and root levels and to decreased tolerance to abiotic stresses such as drought and salinity.

Our results showed a decrease in Chl and carotenoid contents (Table 1), especially at the highest Li concentrations used. Dawood et al. [18] for canola and Hawrylak-Nowak et al. [7] for sunflower and corn described that the Chl content was not significantly affected by Li treatment at low concentrations, although a decrease occurred at the highest concentrations they used. These authors also described an increase in the Chl *a/b* ratio, which contrasted with our results, as well as a decrease in the carotenoid content, similar to that observed in our case. However, for corn, these same authors described a decrease in the Chl content, the Chl *a/b* ratio, and carotenoid content. The fact that the Chl *a/b* ratio was practically unaffected indicates that the capacity of Li-treated plants to efficiently capture light energy is hardly affected by Li. This was also evidenced by similar F_0_ values, which indicated a good photosynthetic state, which was only affected in Li10, where a decrease in F_v_/F_m_ was observed. Li et al. [21] for *B. carinata* described a decrease in the Chl content at concentrations of ≥60 mM, with a more pronounced decrease in the Chl *a* content. Jiang et al. [15] for Li hyperaccumulating in the plant *A. venetum* described a decrease in the Chl and carotenoid contents at concentrations of ≥200 mg kg^−1^, but without any effect on their contents with treatments of 50 mg kg^−1^ Li. Bakhat et al. [41] for spinach observed that low Li concentrations, 20 mg kg^−1^, induced a decrease in the Chl content, while it was not altered when the concentration increased, as was the case for carotenoids at any Li concentration. This unequal response was also described by Shakoor et al. [42] studying soybeans, where they obtained increases in the Chl content at concentrations lower than 50 mg kg^−1^ and decreases at higher concentrations. Only Li10 negatively affected F_V_/F_M_ and altered F_V_/F_0_ (a stress indicator). These results could indicate that the doses of Li used, except Li10, did not significantly alter the fluidity of thylacoids and PSII appression [52]. These results were consistent with the observations by Jiang et al. [15] with the hyperaccumulator *A. venetum*, where Li treatments did not alter the photosynthetic rate despite decreases in the photosynthetic pigment content and stomatal conductance. The damage observed on the leaves, in addition to the decrease in photosynthetic pigment content, may be the result of degradation processes [5,53]. The samples under the highest concentration of Li (Li10) showed a decrease in photosynthetic efficiency (F_V_/F_M_), which is also related to a higher degree of stress, as shown by the F_V_/F_0_ ratio, indicating an alteration in the electron transport process in photosystems. Moreover, Li has the ability to replace Mg in chlorophylls, negatively affecting stomatal conductance and Calvin cycle activity, and to inactivate the photochemical reaction centers of photosystem I (PSI) and photosystem II (PSII) [54].

Li-induced peroxidation appears to depend on the plant and the Li concentrations used. Our results showed similar peroxidation levels in the roots treated with Li as in the controls; however, an increase in the peroxidation level was observed in leaves in all Li treatments (Figure 4A). Hawrylak-Nowak et al. [7] for sunflowers also observed little effect of Li on the peroxidation levels in the roots, but in leaves (50 mg Li kg^−1^) they described an increase of 68%. The results of Dawood et al. [18] for canola leaves were consistent with this increase. However, for corn, Hawrylak-Nowak et al. [7] described the opposite behavior, with a decrease in the peroxidation level in leaves and an increase in the roots. Bakhat et al. [41] described the null effect of Li on peroxidation for spinach leaves. Shakoor et al. [42] obtained similar increases in the peroxidation levels for soybeans in their treatments with 25 mg Li kg^−1^ and 50 mg Li kg^−1^, where higher concentrations induced a sharp increase in the peroxidation level, inducing evidently greater damage. Similar results were obtained with *A. venetum* and *A. cepa* [31,39].

Protein carbonylation, a biomarker of oxidative stress [55], is a consequence of the oxidative stress induced by Li toxicity. The increase in the H_2_O_2_ content induced by Li in the roots caused an increase in protein carbonylation (although not significant), meanwhile in leaves, where the H_2_O_2_ content did not increase at all, protein carbonylation was not altered (Figure 4B). This carbonylation may be part of the cellular response involved in the stress tolerance mechanism [56]. However, there is no evidence of the effect of Li on protein carbonylation in plants.

Li does not affect O_2_^.−^ production in roots, but does cause an increase in leaves in all the concentrations (Figure 5A and Figure 6A,B). There is also a sharp increase in H_2_O_2_ content in roots, but not in leaves, where this content is virtually unchanged (Figure 5B and Figure 6C,D). These results in leaves are similar to the response obtained for spinach by Bakhat et al. [41], who did not observe significant increases in Li-induced H_2_O_2_ content. The increase in the H_2_O_2_ content in roots is similar to those described for heavy metal toxicity [57,58,59]. When increasing the Li content there is an increase in the ROS formation. Although in roots there is no increase in O_2_^.−^ production or SOD activity (Figure 7A), there is a strong increase in H_2_O_2_ content. This increased production may be due to the action of type III peroxidases in the cell wall [60], that are involved in the processes of cell wall reinforcement and the immobilization of the metallic elements in it. On the contrary, in leaves, with much higher amounts of Li in their tissues, the production of O_2_^.−^ and SOD increases, but not H_2_O_2_. Similar increases in the SOD activity have been described for a few species subjected to Li toxicity [13,41,42,51]. In roots, O_2_^.−^ and NO (a decrease in NO content occurs at higher Li concentrations, Figure 6E,F) may be being used to form ONOO^−^, as evidenced by the increase in their content (Figure 6I,J). Furthermore, the increase in the amount of O_2_^.−^ in leaves agrees with the results described by Dawood et al. [18] for canola leaves, who observed how Li causes an increase in O_2_^.−^ content, although unlike our results, they also observed an increase in H_2_O_2_ and a decrease in the SOD activity. Dawood et al. [18] described an increase in NO and O_2_^.−^ along with a decrease in H_2_S content due to the effect of Li in canola leaves. In toxicity induced by V [61] and Cd [57], increases in endogenous NO take place, which in turn positively modulate the SOD activity and reduce the MDA production. These results are consistent with our results for sunflowers. The decrease in H_2_S levels observed in both roots and leaves (Figure 5D and Figure 6G,H) may be due to the ability of Li to react directly with this compound, forming Li_2_S, which might act as a detoxification system [62].

The enzymes of the ascorbate-glutathione cycle (APX, DHAR, MDHAR, and GR) are activated in response to Li in both roots and leaves (Figure 7), depending on the Li concentration to which they are exposed. Similar alterations in APX and POD activities have been described in response to Li [41,42], but there are no references for the other activities of the AsA–GSH cycle. Increases in the contents of H_2_O_2_, ONOO, and NO can modulate the activity of some of these enzymes, such as APX [63,64]. In the roots, Li-induced stress caused an increase in protein carbonylation, while no changes in lipid peroxidation were observed, which was possibly due to the increase in APX activity, which can be modulated by nitrosylation processes [65]. However, in leaves where the O_2_^.−^ content increased and the H_2_O_2_ content was practically unchanged, there was a slight increase in peroxidation, without an alteration in protein carbonylation.

It could be observed in both organs that the AsA content decreased and the DHA content increased (Figure 8A,B), especially at higher Li concentrations. This led to an alteration in redox homeostasis, with decreases in AsA-DHA. However, glutathione levels in the roots decreased (Figure 6M,N and Figure 8C), both the oxidized and the reduced, despite there being a clear increase in GR activity. In leaves (Figure 8D), this content was either unaffected (GSH) or decreased at certain Li concentrations (GSSG). This decrease in glutathione could be due to the fact that part of this compound can be diverted to the formation of phytochelatins or compounds whose purpose it is to eliminate the toxic effects of certain elements, such as Li. Li induced a decrease in the GSH content, with an increase in H_2_O_2_ production. This can inhibit GSNOR activity, thereby inducing an increase in the SNO content, causing a decrease in the GSSG content, and increasing protein nitration processes, thereby modulating the functioning of activities such as APX [66]. The decrease in H_2_S and GSH contents may be due to the affinity of Li for the sulfhydryl group, thus changing its chemical state [67]. Consistent with these results, Dawood et al. [18] showed a decrease in the contents of both reduced ascorbate and glutathione and H_2_S due to high Li concentrations.

Gene expression can be altered by the presence of Li at high concentrations, as Li is able to stabilize or destabilize both DNA and RNA directly, without needing to modify intermediary proteins involved in Li sensing [68]. Regarding the evaluation of gene expression, the expression of *CAT*, *DHAR*, *DHAR_like*, and *GST* was quantified and analyzed, with *CAT* being the only one that showed an expression that increased significantly as the Li concentration increased (Figure 9). It is true that there are studies that have determined a decrease in *CAT* activity, even in plants exposed to low doses of Cd [69,70]. This fact is somewhat contradictory since *CAT*, being an enzyme with great influence on the antioxidant response to ROS, should have high activity due to the oxidative stress generated by the presence of these heavy metals or metalloids [71]. However, this can be explained by the effect of metal toxicity, which affects the functioning of this particular enzyme [72,73]. The results obtained might indicate that, as the amount of Li increases, *CAT* activity is impaired, causing an increase in the oxidative environment, with the plant responding by increasing *CAT* expression. This increased expression may not be reflected in greater activity as it may undergo post-transcriptional or post-translational modifications that prevent its proper functioning.

The other enzymes for which their expression was quantified are two isoforms of *DHAR* and one *GST*. *DHAR* is part of the AsA-GSH cycle, which is key to the detoxification of both H_2_O_2_ and phytotoxic compounds [74,75]. There are a multitude of different *DHAR* isoforms described in the cytoplasm, vacuole, chloroplasts, or peroxisomes of different species [76,77,78,79,80,81]. *Glutathione S-transferase* (*GST*) contributes to the catalytic elimination of ROS and xenobiotic compounds through GSH, regulating the redox balance and maintaining the basic functions of ROS signaling [82]. The *GST* gene family exhibits broad multifunctionality and functional complexity; therefore, exhaustive genome-wide analyses have been conducted on several species [83,84,85,86,87].

The relationship between *DHARs* and *GSTs* lies in the fact that, although *DHARs* cannot catalyze *glutathione S-conjugation* or peroxide reduction, they are considered to be members of the *GST* superfamily [88]. This is despite the fact that *DHAR*, rather than stabilizing the thiolate anion of GSH (GS–), reversibly forms disulfide bonds with GSH as part of the catalytic mechanism [89].

*DHAR* plays a key role in the regulation of AsA and GSH [74,90]. The main *DHAR* isoforms studied are those with cytosolic and chloroplastidial localization due to their prominent participation in maintaining the redox state [91], especially under environmental stress [90]. This role has been confirmed in overexpression experiments [90]. In *Solanum tuberosum*, through overexpression of *StDHAR1* and *StDHAR2* (cytosolic and chloroplast *DHAR*, respectively), an increase in the AsA content in leaves and tubers was shown with the first isoform and a greater accumulation of AsA only in leaves was shown with the second isoform [92,93]. This indicated that cytosolic *DHAR* can regulate AsA levels in different organs but that the regulatory capacity of the chloroplast isoform might be limited to photosynthetic tissues. In our case, there is no literature about these isoforms, but it seems that *DHAR* is more involved in defense against Li at the root level than in leaves, since its expression level in leaves decreased when facing stress but increased, very significantly, in the roots depending on the amount of Li. This increase stabilized in Li7.5, with the expression pattern being very similar in Li 10. The expression of *DHAR_like*, on the other hand, significantly increased at the leaf and root levels, but it seemed to saturate in Li5 in leaves and Li7.5 in the roots, so its expression did not stabilize in the same manner as *DHAR* in the roots but rather decreased significantly. These data were consistent with the *DHAR* activity detected in both the roots and leaves, which showed an increase in activity with Li concentration, although at high Li concentrations this activation disappeared. Further evaluation of these isoforms is necessary to discover possible domains that give some isoforms greater importance in plant defense against abiotic stress and their possible localization, as well as their regulation at the levels of expression and activity.

*GST*, like *DHAR*, uses GSH to carry out its function. The *GST* isoform analyzed showed an increase in its expression level with each increase in Li concentration, up to Li10, where there was a pronounced and significant decrease in its expression, although it was significantly higher than that quantified in the roots of control plants. Li at this concentration (10 mM) could be considered to saturate the capacity of *GST* to form Li conjugates with GSH, or the low amount of free GSH means that an increase in the transcription of the genes encoding this *GST* is not necessary, but that there is more free GSH. This result could explain why the expression of *DHAR* and *DHAR_like* in the roots in Li10 increased slightly or did not decrease excessively, respectively. If there is more expression of these genes encoding *DHAR* and *DHAR_like*, this would imply greater activity of both isoforms, which, in this way, would translate into a high consumption of GSH due to the oxidative stress that the plant is suffering, thus not leaving any free GSH for the detoxifying action of enzymes such as *GST* (as can be observed in the sharp decrease in the GSH content in Li10, Figure 8C). In fact, some studies show that, although GSH oxidation is potentially mediated by some *GSTs* and peroxiredoxins (PRXs) [94], *DHAR* is an important factor in ensuring GSH oxidation during oxidative stress [81,95].

Regarding epigenetic studies, DNAg methylation has been analyzed by determining the transfer of methyl groups from the C5 position to cytosine, forming 5-methylcytosine (5mC) [96]. In plants, DNA methylation occurs in three sequence contexts: CG, CHG, and CHH (where H = A, C, or T). Methylation at CG and CHG, both symmetric, is maintained during replication by the methyltransferases MET1 and CMT3, respectively, and is associated with gene regulation and the silencing of transposable elements [97,98]. By contrast, methylation at CHH, which is asymmetric, is established de novo by DRM2 through the RNA-directed pathway (RdDM), involving small RNAs, and is maintained in heterochromatic regions by CMT2 [98]. These three types of methylation work together to control gene expression, protect genome integrity, and respond to environmental signals [98]. Furthermore, DNA methylation is not limited to the promoter regions of genes but can also occur in the coding regions [97,98].

There are numerous examples of changes in DNAg methylation in plants when they are exposed to high doses of xenobiotic compounds, such as heavy metals or metalloids. Some studies reported a decrease in DNAg methylation or hypomethylation in individuals exposed to these type of stresses. Choi et al. [99] described DNA demethylation in *Nicotiana tabaccum* cv Xanthi nc plants as a consequence of stress caused by high Al concentrations. Along the same lines, Ou et al. [100] observed, under Cu toxicity, hypomethylation in *Oryza sativa* in the CHG context of DNA, without affecting CG or CHH. Furthermore, this epigenetic modification not only existed in plants that were directly treated but seemed to have been inherited by successive generations, thus conferring to the offspring a greater tolerance to that same stress. Other studies mention the hypermethylation of DNAg in response to stress generated by metals at harmful concentrations. Thus, Yagci et al. [101] determined that there was an increase in DNAg methylation in *Lactuca sativa* under Cu stress, which could be reduced by applying NO, therefore stabilizing the genome and mitigating the adverse effects of heavy metal stress. Ertürk et al. [102] analyzed the response of *Zea mays* plants to increasing concentrations of Cu and Mn, describing an increase in DNA methylation, accompanied by a reduction in levels of growth-promoting phytohormones (gibberellic acid, zeatin, and indoleacetic acid). This is logical since if cell growth and division are induced, DNAg demethylation should increase, as well as levels of abscisic acid (ABA), a hormone related to the stress response. However, McKergow et al. [103] did not detect significant changes in DNA methylation in *Picea glauca* subjected to Cu toxicity.

It is possible that, in toxic concentrations of Li, gene expression in the roots is limited to those genes that encode proteins involved in the defense against this stress, while at the leaf level, with lower methylation, there is greater expression of genes involved in both plant development and defense. In Li10, as the methylation was reversed (it was greater in leaves than in the roots), it is possible that the Li accumulated in the aerial parts subjected these organs to significant stress, meanwhile in the roots, the effect was such that they could no longer respond effectively, and there was no regulation of expression observed at concentrations from Li2.5 to Li7.5.

Our results (Figure 10) showed that Li induced hypomethylation in leaves and significant hypermethylation in the roots in Li2.5 and Li5, while in Li7.5 the trend persisted but was not statistically significant. At the highest dose (Li10), methylation in the roots declined, consistent with findings in rice under Cu toxicity, where the highest Cu concentration also caused DNA hypomethylation [104]. These observations suggest that Li stress may trigger hypomethylation in genomic regions linked to tolerance while promoting hypermethylation in less critical regions, with the overall methylation balance depending on the genomic context [105]. Epigenetic regulation clearly plays a role in plant responses to heavy metal stress [106]. Rather than global methylation, it is the methylation status of stress-responsive loci that appears to be decisive. Evidence from *A. thaliana* and rice supports this: demethylation increased the expression of Cu transporters and chelating proteins, enhancing Cu tolerance [107], while histone acetylation and methylation adjustments under As and Cd stress respectively improved detoxification and tolerance [108,109,110]. Identifying the genomic regions underpinning these mechanisms remains a key challenge, particularly given the high inter- and intraspecific epigenetic variability reported under similar stress conditions [111].

## 4. Materials and Methods

### 4.1. Plant Material and Growing Conditions

Seeds of *Helianthus annuus* were surface-sterilized for 15 min in a 10% sodium hypochlorite solution (40 g L^−1^), rinsed several times with distilled water, and before their germination were imbibed in distilled water, aerated, and agitated for 2 h at room temperature. After imbibition, the seeds were germinated in a plastic container (30 × 20 × 10 cm) filled with a sterilized perlite mixture substrate wetted with Hoagland solution at 27 °C in the dark for 48 h. After germination, the seedlings were cultivated for five days at 27 °C with 85% relative humidity and constant illumination under photosynthetic photon flux density (350 μmol m^−2^ s^−1^).

After 7 days, the plants were grown in hydroponic culture in lightweight polypropylene trays (20 × 15 × 10 cm; 4 plants per container) and the same environmental conditions (except for relative humidity, 50%). The plants were treated with a basal nutrient solution composed of 4 mM KNO_3_, 3 mM Ca(NO_3_)_2_ 4H_2_O, 2 mM MgSO_4_ 7H_2_O, 6 mM KH_2_PO_4_, 1 mM NaH_2_PO_4_ 2H_2_O, 10 μM ZnSO_4_ 7H_2_O, 2 μM MnCl_2_ 4H_2_O, 0.25 μM CuSO_4_ 5H_2_O, 0.1 μM Na_2_MoO_4_ 2H_2_O, 10 μM H_3_BO_3_, and 20 μM NaFeIII-EDTA. For the Li treatment, the basal solution was supplemented with LiCl to final concentrations of 0 mM (control, Li0), 2.5 mM (Li2.5), 5 mM (Li5), 7.5 mM (Li7.5), and 10 mM (Li10). Each cultivation solution was adjusted to pH 5.8, continuously aerated, and changed every 4 days. The plants were exposed to Li for 14 days.

Plants from each treatment (4 plants per treatment, 5 independent experiment) were divided into roots and shoots, which were washed with distilled water, dried on filter paper, measured, and weighed to obtain the fresh weight (FW). For each treatment, the roots and shoots were dried in a forced air oven at 70 °C for 48 h to obtain the dry weight (DW) and the subsequent analysis of the concentrations of Li and other mineral elements. To determine the relative growth rate (RGR), shoots (shoots RGR) and roots (roots RGR) from each treatment were sampled at the beginning (T_i_ = 0 days, DW_i_) and end (final DW, DW_f_) of the Li treatments, and the respective RGR was calculated according the expression: RGR = (ln DW_f_ − ln DW_i_)/(T_f_ − T_i_) [112]. Furthermore, the Li tolerance index (LiTI) was also estimated using the expression: LiTI = DW of Li treated plants/DW of control) × 100 [113]. For roots LiTI and shoots LiTI, the value was calculated using the respective DW. Other roots and leaves were used for biochemical and molecular analyses.

### 4.2. Germination Test

Seeds of *Helianthus annuus* were surface-sterilized for 15 min in a 10% sodium hypochlorite solution (40 g L^−1^), rinsed several times with distilled water, and before germination were imbibed in distilled water, aerated, and agitated for 2 h at room temperature. After imbibition, the seeds were germinated in petri dishes filled with sterilized distilled water (control) and with different LiCl concentrations, at 27 °C in the dark for 48 h. Afterward, the germination percentage was measured. Additionally, the germination index (GI) was calculated according to the following formula [114]: GI = (G/G_0_) × (L/L_0_) × 100 (G = number of seeds germinated in Li; G_0_ = number of seeds germinated in the control group; L = mean root length in Li treatment; L = mean root length in the control group).

### 4.3. Determination of Leaf Areas

Photographs of the leaves of 10 different plants for each treatment were taken using a DXM 1200C camera and each photograph was analyzed using the free software Fiji ImageJ 2.3.2. The area was calculated using the polygon and measure tools and all the of leaves of each plant were considered for the total area.

### 4.4. Elemental Content Analysis

The plant material, including roots and shoots from the control and Li treatments, was harvested and rinsed with distilled water. After 72 h of drying at 70 °C, the root and leaf material was crushed in a ceramic mill. The Li, Na, K, Ca, Mg, Fe, Mn, copper (Cu), and Zn contents were measured using inductively coupled plasma mass spectrometry (ICP-MS, model NexION300, PerkinElmer, Shelton, CT, USA), following the method described by Lehotai et al. [115]. The bioaccumulation factor of Li (BCF_Li_) was calculated from the ratio between the concentration of the element in the roots or shoots and that present in the hydroponic solution, and the translocation factor (TF_Li_) was calculated from the ratio between the concentration of this element in the shoots and the roots.

### 4.5. Chlorophyll and Carotenoid Contents and Photochemical Efficiency

The chlorophyll (Chl) and carotenoid contents of the leaves were determined at the end of each trial. About 0.125 g of fresh leaves was homogenized in 5 mL of 80% acetone, then 5 mL more was added and then centrifuged for 10 min at 1200× *g*. The concentrations of chlorophyll *a* (Chl *a*), chlorophyll *b* (Chl *b*), and carotenoids were measured spectrophotometrically at 663, 646, and 470 nm. The total Chl and carotenoid contents were calculated following the method described by Wellburn [116].

To determine the photosynthetic parameters, the middle region of the fully expanded upper leaves at the end of each Li treatment were adapted in the dark for 10 min, and then the minimal fluorescence (F_0_), the maximal chlorophyll fluorescence (F_M_), and the maximum photosynthetic efficiency (F_V_/F_M_) were recorded using a handheld fluorometer (Chlorophyll Fluorometer, OS-30p, Opti-Sciences, Hudson, NH, USA). The variable fluorescence (F_V_) and the rate constants of photochemical and nonphotochemical deactivation of excited Chl molecules (F_V_/F_0_) were calculated.

### 4.6. Oxidative Stress Analysis

To analyze the oxidative damage to lipids, the formation of malondialdehyde (MDA) was determined to be the indicator of lipid peroxidation using thiobarbituric acid (TBA). Briefly, 0.25 g of plant material (leaves or roots) was homogenized with 2.5 mL of solution containing 0.25% TBA and 10% TCA. The mixture was incubated at 95 °C for 30 min. The reaction was stopped by immersing the tubes in ice, filtering, and centrifuging them at 8800× *g* for 10 min. The MDA content was determined in the supernatant at 532–600 nm. The MDA concentration was calculated using ε = 155 mM^−1^ cm^−1^ and expressed as μmol MDA g^−1^ FW [117].

The oxidative damage to protein was estimated from the content of carbonyl groups [118]. The leaves or roots were homogenized in 100 mM phosphate buffer, 0.1% Triton, 1 mM EDTA, 0.1 mM PMSF, 1 g L^−1^, pH 7.0 (0.05 or 1 g mL^−1^, respectively). The homogenized material was filtered and centrifuged at 1300× *g* for 20 min at 4 °C. The carbonyl content was determined in the supernatant through its reaction with 2,4 dinitrophenyl hydrazine (DNPH) in 2 M HCl for 1 h. The reaction was stopped with 20% TCA and the mixture was centrifuged at 11,000× *g* for 3 min. The pellets were washed 2 or 3 times with ethanol-ethyl acetate (1:1). The precipitated protein was dissolved in guanidine 6 M, incubated at 37 °C for 30 min, centrifuged at 11,000× *g* for 3 min. The carbonyl group concentration was determined at 370 nm using ε = 22,000 M^−1^ cm^−1^ and expressed as nanomole mg^−1^ protein.

O_2_^.−^ generation was measured in the plant material (leaves or roots, 0.5 g mL^−1^), which was homogenized at 4 °C in 50 mM phosphate buffer, 0.5 mM PMSF, 1 g L^−1^ PVPP, 1 mM mercaptoethanol, pH 6.8. The homogenate was filtered and centrifuged at 39,000× *g* for 30 min at 4 °C, and the supernatant was collected as an enzyme extract. O_2_^.−^ generation was assayed spectrophotometrically by measuring the oxidation of epinephrine to adrenochrome at 480 nm (ε = 4.020 mM^−1^ cm^−1^) [119]. The reaction mixture contained 1 mM epinephrine in 50 mM phosphate, pH 6.8.

Furthermore, the content of H_2_O_2_ was analyzed following the method described by Velikova et al. [120]. Fresh leaves (0.2 g mL^−1^) or roots (0.3 g mL^−1^) were homogenized in 0.1% trichloroacetic acid (TCA). The homogenate was centrifuged at 12,000× *g* for 15 min, and the reaction mixture contained 0.5 mL supernatant, 0.5 mL 10 mM potassium phosphate buffer (pH 7.0), and 1 mL of 1 M KI solution. The H_2_O_2_ concentration was estimated based on the absorbance of the reaction mixture at 390 nm using a standard curve of H_2_O_2_.

The nitric oxide (NO) content was determined using the method described by Zhou et al. [121]. Fresh leaves or roots were homogenized in 3 mL of 50 mM cool acetic acid buffer (pH 3.6, containing 4% zinc diacetate). The homogenates were centrifuged at 10,000× *g* for 15 min at 4 °C. The supernatant was collected. The pellet was washed using 1 mL of extraction buffer and centrifuged as before. The two supernatants were combined and charcoal was added, then they were vortexed and filtered. The mixture of filtrate and Griess reagent (1:1) was incubated at room temperature for 30 min. Absorbance was determined at 540 nm. The NO content was calculated by comparison to a standard curve of NaNO_2_.

The H_2_S concentration was determined following the method described by Li [122]. Fresh material was homogenized in 20 mM Tris-HCl buffer pH 8.0 containing 10 mM EDTA and 20 mM Zn(OAc)_2_ (0.5 g mL^−1^) and centrifuged at 15,000× *g* for 15 min at 4 °C. The supernatant was combined with 30 mM FeCl_3_ (in 1.2 M HCl) and 20 mM DMPD (in 7.2 M HCl) (1:1:1). The mixture was incubated at room temperature for 15 min and the absorbance was determined at 670 nm. The H_2_S content was calculated by comparison to a standard curve of NaHS.

### 4.7. Determination of Antioxidant Enzymes and Components of the AsA–GSH Cycle

Superoxide dismutase activity (SOD, EC 1.15.1.1) was determined as the absorbance at 560 nm in 50 mM phosphate buffer pH 7.8, 0.1 mM EDTA, 1.3 μM riboflavin, 13 mM methionine, and 63 μM NBT [123], following enzyme extraction (see extract enzyme for O_2_ determination). A unit of SOD was defined as the amount of enzyme required to cause 50% inhibition of NBT reduction.

To determine the activities of the enzymes involved in the AsA–GSH cycle—APX, MDHAR, DHAR, and GR—the roots or leaves (0.75 or 0.5 g L^−1^, respectively) were homogenized at 4 °C in 50 mM phosphate buffer, pH 7.5, 0.5 mM PMSF, 1 mM β-mercaptoethanol, 1 g L^−1^ PVPP, and 5 mM AsA for APX activity. The homogenate was filtered and centrifuged at 3900× *g* for 30 min at 4 °C, and the supernatant was used for the enzyme determinations. The APX activity was determined spectrophotometrically by measuring the oxidation of ascorbate at 290 nm for 2 min (ε = 2.8 mM^−1^ cm^−1^) [124]. The reaction mixture contained 0.5 mM ascorbate, 0.2 mM H_2_O_2_, and the enzyme extract, at 25 °C, in 0.1 M phosphate buffer, pH 7.5, and 0.5 mM EDTA, with the result expressed as μmol ascorbate min^−1^ mg^−1^ protein. The DHAR activity was determined from the oxidation of DHA at 265 nm for 1 min (ε = 14 mM^−1^ cm^−1^) [125] in a medium containing 0.1 M phosphate buffer (pH 6.5), 0.5 mM EDTA, 2.5 mM GSH, 0.5 mM DHA, and the enzyme extract. The DHAR activity is expressed as nmol ascorbate min^−1^ mg^−1^ protein. The MDHAR activity was determined from the oxidation of NADH at 340 nm for 1 min (ε = 6.22 mM^−1^ cm^−1^) [126] in a medium containing 50 mM Tris-HCl buffer (pH 7.8), 10 mM AsA, 0.2 mM NADPH, 0.5 units of ascorbate oxidase, and the enzyme extract, with the result expressed as μmol NADH min^−1^ mg^−1^ protein. The GR activity was determined at 340 nm from the oxidation of NADPH for 3 min (ε = 6.22 mM^−1^ cm^−1^) [125] in a medium containing 0.1 M phosphate buffer (pH 7.5), 0.5 mM EDTA, 0.5 mM GSSG, 0.2 mM NADPH, and the enzyme extract, with the result expressed as nmol NADPH min^−1^ mg^−1^ protein.

For the total ascorbate and glutathione, fresh roots or leaves (0.625 or 0.25 g mL^−1^, respectively) were homogenized at 4 °C in 5% sulfosalicylic acid. The homogenate was centrifuged at 20,000× *g* for 20 min at 4 °C, and the supernatant was collected for the determination of ascorbate and glutathione. The total ascorbate pool (AsA + DHA) and total glutathione pool (GSH + GSSG) were determined following the method described by De Pinto et al. [127]. The total ascorbate was determined by the reduction of DHA to AsA, and the concentration of DHA was estimated by the difference between the total ascorbate pool and AsA. The ascorbate pool was determined at 525 nm. The glutathione pool was determined by the change in absorbance at 412 nm over 1 min. GSH was estimated as the difference between the amount of the total glutathione pool and that of GSSG.

### 4.8. Visualization and Determination of ROS, RNS, H_2_S, and GSH

Control and Li-treated plant roots (20 mm) were incubated in the dark for 30 min at 37 °C with 10 µM DHE (for O_2_^.−^ detection) or 25 µM DCF-DA (for H_2_O_2_ detection) in 10 mM Tris-HCl, pH 7.4, then rinsed with the same buffer thrice for 15 min each time [128]. For the determination of NO and ONOO^−^, the roots were incubated in the dark for 60 min at 25 °C with 10 µM DAF-2DA (for NO detection) or 10 µM APF (for ONOO^−^ detection) in 10 mM Tris-HCl, pH 7.4. They were then rinsed with the same buffer thrice for 15 min each time [128]. To detect RSNOs, the intact root samples were incubated with 10 mM NEM in the dark for 60 min at 25 °C, and rinsed thrice with 10 mM Tris-HCl, pH 7.4 (for 15 min each time). Then they were incubated for 60 min in the dark at 25 °C with 10 µM Alexa-Fluor 488 Hg-link phenylmercury [129]. Finally, they were rinsed thrice with the same buffer (for 15 min each time). To detect H_2_S, the roots were incubated with 100 µM 7-azido-4-methylcoumarin (AzMC) for 40 min in 10 mM phosphate buffer (pH 7.4) at room temperature in the dark [130], and then rinsed thrice with the same buffer (for 15 min each time). To detect GSH, the roots were incubated for 30 min in 50 µM monochlorobimane (MCB) in 10 mM phosphate buffer (pH 7.2) at room temperature in the dark [131], and then rinsed thrice with the same buffer (for 15 min each time). Finally, the whole (non-fixed) roots were placed on a slide for examination under fluorescence microscopy (Axioplan-Zeiss microscope, Oberkochen, Germany). In each case, λ_exc_ and λ_em_ were adjusted to the respective probe. At least 10 roots were tested under each experimental condition, and five independent replicates were analyzed. The ImageJ program was used to process and analyze the images, and the fluorescence intensity was expressed in arbitrary units.

### 4.9. DNAg Extraction and Cytosine Methylation Study

To determine the level of cytosine methylation in *H*. *annuus* samples, DNAg was extracted from samples from the control group and those exposed to Li (Li2.5, Li5, Li7.5, and Li10). Extraction was performed following the protocol proposed in the “Plant/Fungi DNA Isolations Kit” (Norgen Biotek Corp., Thorold, ON, Canada). DNAg was quantified and its quality was assessed using a biospectrophotometer “spectrometer” (Eppendorf AG, Hamburg, Germany). The quality was assessed based on the 260/280 ratio, and the quantity was quantified in μg mL^−1^. The samples used were those with a ratio between 1.7 and 2.1. Sample integrity was subsequently assessed using 1% agarose electrophoresis (Condalab, Madrid, Spain) in SYBR™ Safe DNA Gel Stain in 1X TAE (Thermo Fisher Scientific, Waltham, MA, USA), and the gel was developed using a Geneflash transilluminator (SynGene, Cambridge, UK).

DNAg samples with sufficient concentration (between 50 and 100 μg/mL), quality, and integrity were analyzed in duplicate to assess the cytosine methylation level using the MethylFlash™ Methylated 5m cDNA—Quantification Kit (EpigenTek, Farmingdale, NY, USA) and a microplate photometer (Biosan, Riga, Latvia). The calculation of absolute 5-mC (ng) values used the formula: 5-mC (ng) = (Sample OD − ME3 OD)/(Slope × 2*).

### 4.10. RNA Extraction and Transformation into cDNA

RNA was extracted and purified from the leaf organs of different whorls (from young to adult leaves) and lateral root fragments after 14 days of being exposed to Li. The plant material was harvested, homogenized, and stored in a chamber at −80 °C until extraction. The extraction protocol followed that indicated in the “Spectrum Plant Total RNA Kit” (Thermo Fisher Scientific, Waltham, MA, USA). Once purified, its concentration, quality, and integrity were assessed. The first parameters were measured using a biospectrophotometer “spectrometer” (Eppendorf AG, Hamburg, Germany), where the 260/280 ratio was calculated, and only the samples in the range of 1.7–2.1 were used. Integrity was assessed on a 1% agarose electrophoresis gel (Condalab, Madrid, Spain) in SYBR™ Safe DNA Gel Stain in 1X TAE (Thermo Fisher Scientific, Waltham, MA, USA), and the gel was developed on a Geneflash transilluminator (SynGene, Cambridge, UK).

Each RNA sample was reverse-transcribed into cDNA using the “High Capacity cDNA Reverse Transcription Kit” (Applied Biosystems, Foster City, CA, USA) and the T100 thermal cycler (Bio-Rad, Hercules, CA, USA).

### 4.11. qPCR Analysis

Quantification of catalase expression (*CAT*), *DHAR*, *DHAR_like*, and *GST* genes was performed using the SYBR Green kit (Applied Biosystems, Foster City, CA, USA) and the Applied Biosystems QuantStudio 1 thermal cycler (Thermo Fisher Scientific R, Waltham, MA, USA). The primers used are shown in Table 2 and Table S1. Actin was used as housekeeping to normalize the results of the expression of the genes under study.

### 4.12. Statistical Analysis

More than 15 independent biological samples per treatment were analyzed, with at least three technical replicates performed for each sample for each parameter evaluated. Phenotypic and biochemical parameters, gene expression quantification, and methylation determination were subjected to a Shapiro–Wilk test to assess whether the obtained values followed a normal distribution; subsequently, a one-way ANOVA was performed to determine whether significant differences existed among samples from the different treatments. These statistical analyses were carried out using RStudio version 4.3.2 (RStudio, Inc., Boston, MA, USA), and statistically significant differences (*p* < 0.05) are represented by letters. Graphs were generated using Microsoft Excel (MicrosoftR Corp., Alburquerque, NM, USA).

## 5. Conclusions

Our study provides evidence of how stress induced by different concentrations of Li negatively affects sunflower germination, growth, and biomass. The absorbed Li accumulate mainly in the shoots. Li toxicity induces alterations in mineral nutrient homeostasis (K, Na, Fe, and Cu). No alterations in the photosynthetic pigment content or photosynthetic efficiency were observed, only decreases in Li10. Li toxicity induces stress, which is reflected through increases in Li peroxidation and protein carbonylation, with altered production of ROS and H_2_S, but not NO. The enzymatic antioxidant system is altered, increasing its activity, as well as cellular redox homeostasis. Li appears to interact with SH groups, as reflected by decreases in H_2_S and GSH levels. The quantification of the expression of *CAT*, *DHAR*, *DHAR_like,* and *GST* revealed the influence of these enzymes in the plant’s defense against stress caused by an increase in Li concentration. It is necessary to continue studying the genetic regulation of these and other defense genes. Similar to the enzymatic activities, the expression of the two *DHAR* isoforms analyzed differed depending on the organ studied and the Li concentration. Likewise, the regions that are activated and deactivated to respond to stress through epigenetic mechanisms (methylation/demethylation of DNAg regions) should be studied in depth because there were observed changes in trends in relation to the percentage of DNAg methylation in response to the different doses of Li supplied to sunflower plants. Li induces lipid peroxidation and enhances the generation of O_2_·^−^. In response to these oxidative challenges, the antioxidant enzymes of the AsA–GSH cycle exhibit increased activity in proportion to the Li concentration. At the foliar level, where Li accumulation is markedly higher than in the roots, these enzymatic activities effectively regulate H_2_O_2_ levels, which remain largely unaltered. This suggests the activation of an efficient antioxidant defense mechanism against Li-induced oxidative stress, particularly at higher Li concentrations, where the extent of lipid peroxidation is comparable to that observed at lower concentrations. AsA and GSH contents generally decline, except for foliar GSH, which remains stable. In this context, the observed upregulation of *GST* expression is particularly noteworthy, as it is an enzyme that is closely linked to GSH metabolism and has a dual role as an antioxidant and as an Li-chelating molecule. Collectively, these findings indicate that Li exerts a significant influence on both the activity and expression of antioxidant enzymes, thereby modulating redox homeostasis under Li exposure.

## Figures and Tables

**Table 1:** Effect of Li on chlorophyll a and b contents (µg g^−1^ FW), carotenoid content (µg g^−1^ FW), chlorophyll a/b and carotenoids/total chlorophyll ratios, and photosynthetic parameters (F_0_, F_M_, F_V_, F_V_/F_M_, and F_V_/F_0_) of *H. annuus* leaves.

**Treatment**	**Chl a**	**Chl b**	**Chl a/b**	**Car**	**Car/Chl**
Li0	1470.2 ± 51.3 b	472.1 ± 24.6 b	3.13 ± 0.05 a	439.0 ± 13.7 b	0.227 ± 0.004 a
Li2.5	1362.9 ± 37.2 b	412.3 ± 13.6 ab	3.31 ± 0.06 a	410.7 ± 18.2 ab	0.223 ± 0.004 a
Li5	1226.4 ± 134.7 ab	390.6 ± 52.9 ab	3.11 ± 0.09 a	376.4 ± 35.2 ab	0.235 ± 0.009 a
Li7.5	1063.7 ± 82.8 a	328.8 ± 23.8 a	3.23 ± 0.07 a	327.2 ± 24.5 a	0.236 ± 0.011 a
Li10	1029.4 ± 64.6 a	317.6 ± 11.5 a	3.23 ± 0.09 a	310.6 ± 18.2 a	0.230 ± 0.003 a
**Treatment**	**F_0_**	**F_M_**	**F_V_**	**F_V_/F_M_**	**F_V_/F_0_**
Li0	270.0 ± 2.8 a	1539.8 ± 20.8 b	1259.2 ± 27.4 b	0.817 ± 0.008 b	4.54 ± 0.21 b
Li2.5	260.8 ± 7.3 a	1552.5 ± 18.2 b	1275.6 ± 10.3 b	0.822 ± 0.008 b	4.46 ± 0.28 b
Li5	267.4 ± 3.2 a	1502.0 ± 30.7 ab	1222.1 ± 30.5 ab	0.813 ± 0.006 ab	4.41 ± 0.22 b
Li7.5	269.3 ± 4.5 a	1512.8 ± 43.8 ab	1242.7 ± 44.9 ab	0.821 ± 0.007 b	4.63 ± 0.19 b
Li10	278.8 ± 3.7 a	1386.7 ± 44.7 a	1103.1 ± 50.8 a	0.761 ± 0.023 a	3.49 ± 0.35 a

Values are means, error bars denote ± standard deviation (n = 15), and different letters indicate a significant difference among treatments (*p* < 0.05).

**Table 2:** Oligonucleotides used for real-time quantitative RT-PCR analysis of *Actin*, *CAT*, *DHAR*, *DHAR_like*, and *GST*.

Gene	Forward Primer	Reverse Primer	Size (bp)
*Actin*	AGCTGCTGGTATTCACGAGACC	TCGATCCTCCGATCCAGACACTG	224
*CAT*	ACACTCAGAGGCACCGTCTT	GCTTTCCGGACAACCTAACA	201
*DHAR*	ATGGGACTGAACAGGCTTTG	AACGGCTGTGACCTTTTCTC	98
*DHAR_like*	TTTGAGTCTGGCACCAAAGC	TTTCGGGAACAGTCCACTTC	74
*GST*	GAAAATCCCGGTTCTCATCC	ATCAGAGGGGAACAATGGAG	109

## Data Availability

Data are contained within the article.

## Supplementary Materials

The following supporting information can be downloaded at: https://www.mdpi.com/article/10.3390/plants15030421/s1, Table S1. Information about primers used for amplification of sunflower cDNA [132,133].
