# Supplementary material for: Effects of High Lithium Concentrations on the Growth, Biomass, Mineral Accumulation, Oxidative Stress, Antioxidant and Gene Expression Response, and DNA Methylation in Sunflower Plants
Source: Plants (Basel). 2026 Jan 30;15(3):421. doi: 10.3390/plants15030421 (PMC12899685; doi:10.3390/plants15030421)
Supplement: Supplementary file 1 [file plants-15-00421-s001.zip › plants-4116489-Table S1.pdf]

Table S1. Information about primers used for amplification of sunflower cDNA

Gene	Accession numbers/Location	Information/References
Actin	CA254672	[132]
Catalase	L28740/AF243517/AF243518/AF243519	[132]
DHAR	HanXRQr2_Chr08g0332021	>HanXRQr2_Chr08g0332021:mRNA:HanXRQr2_Chr08g0332021 cds:protein_coding
DHAR_like	HanXRQr2_Chr12g0541051	>HanXRQr2_Chr12g0541051:mRNA:HanXRQr2_Chr12g0541051 cds:protein_coding
GST	KR071872/HanXRQr2_Chr10g0452211	[133]
